# Enhanced Recovery Pathway in Adults Undergoing Elective Posterior Thoracolumbar Fusion Surgery: Outcomes Compared with a Traditional Care Pathway

**DOI:** 10.1155/2021/6204831

**Published:** 2021-09-15

**Authors:** Khalid AlSaleh, Khalid Murrad, Abdulmajeed AlZakri, Osama Alrehaili, Waleed Awwad

**Affiliations:** ^1^Department of Orthopedics, College of Medicine, King Saud University, Riyadh, Saudi Arabia; ^2^College of Medicine, King Saud University, Riyadh, Saudi Arabia

## Abstract

**Introduction:**

Spine fusion surgery is an increasingly popular procedure, but the patient experience is variable and the cost is high. Enhanced recovery after surgery (ERAS) pathways can provide a standardized plan for spine fusion cases, improving quality of care and reducing costs. We report an early attempt at the implementation of such a pathway and compare it to a historical cohort.

**Methods:**

All adult patients undergoing elective posterior thoracolumbar spine fusion in 2019 and 2020 were included in the study. The ERAS protocol implementation started in January 2020. The study cohort was all cases performed in 2020—after implementation of ERAS—while the historical cohort was cases from 2019. Demographic and clinical data were collected and compared between the groups.

**Results:**

Ninety-three patients were included in the study. The study cohort (ERAS) included 42 patients, while the comparison group (pre-ERAS) included 51 patients. Demographic and preoperative clinical data were similar between the two groups. However, postoperative clinical data showed that ERAS resulted in less reliance on analgesics, earlier mobilization, and a reduced length of stay. Complication and readmission rates were unchanged.

**Conclusion:**

ERAS can reduce costs while maintaining or improving clinical outcomes for spinal fusion surgery.

## 1. Introduction

Globally, spinal fusion surgery rates have increased 54–62% alone in the past decade [1–4]. The complexity of the procedures and the average age of the patient have also increased. This leads to concerns about increased complication rates, reoperation rates, and cost. Standardized clinical care pathways have been shown to reduce costs and improve the quality of care in orthopedic surgery [5, 6]. This was achieved by reducing the variability in perioperative management according to evidence-based recommendations. This variability exists in spine surgery, as shown in recent reports [7, 8]. These reports outline that the more complex the spinal procedure is, the more variability exists in costs and clinical outcomes between different centers. Rooted in early attempts to reduce the surgical stress response, an “enhanced recovery after surgery” protocol (ERAS) has evolved to its current status as a standardized, comprehensive, multidisciplinary approach to perioperative care. This approach typically includes preoperative counseling, management of analgesia, nutrition, wound care, postoperative rehabilitation, and complication avoidance [9]. Regular auditing and reporting on measurable outcomes—such as length of stay, pain scores, and overall procedure cost—helps refine further and improve the protocol to achieve the desired results. ERAS protocols have since been applied to spine surgery, mostly in cases of spinal decompression or minimally invasive surgery [10, 11]. In our institution, we utilized a standardized clinical pathway for spine fusions for several years. The pathway was mainly based on a traditional “standard of care,” and regular auditing was not enforced. Increased awareness about ERAS helped gain institutional support for its implementation in various surgical specialties, including spine surgery. Our goal was to perform a preliminary study—for one year—by implementing a new ERAS pathway for spine fusion surgery in the year 2020. The results of the preliminary study will be compared with the traditional pathway to assess its effectiveness. Lessons learned will also be used to build and implement a definitive ERAS pathway in 2021.

## 2. Materials and Methods

The ERAS protocol was prepared and finalized in multidisciplinary group meetings—involving orthopedic spine surgeons, nurses, physical therapists, and anesthesiologists—over 8 weeks in late 2019. The protocol was then finalized, and the plan to start implementation was the first of January 2020. The protocol highlights included: preoperative patient education, perioperative blood-loss minimization strategies, multimodal analgesia, early mobilization, and strict adherence to strategies for prevention of complications such as thromboembolic events and postoperative infection (Table 1). The research ethics board approval was obtained (21/0277/IRB) prior to the collection of the data. All adult cases undergoing elective thoracolumbar fusion surgery in 2020 were included in the study. The comparison group comprised the same cases done the previous year (2019). Patients who underwent the procedure for pathology other than degeneration— for example, infection or trauma—were excluded from both the groups. The data were collected by an independent third party from the electronic medical records (EMR) and stored on a password-protected computer within the institution after anonymizing patient identifiers. The data collected included demographic data as well as operative data (procedure type, number of levels fused, estimated blood loss, length of surgery, and insertion of surgical drain), day of mobilization, day of initiation of oral analgesia, day of urinary catheter removal, day of discharge (DOD), pain score on DOD, analgesic use on DOD, glycemic control on DOD, and rates of complications, reoperations, or readmissions. Categorical and ordinal data were expressed in frequencies and analyzed using the chi-square test and the Mann–Whitney *U*-test, respectively. Parametric data were expressed as means and standard deviations (SD) and analyzed using the independent samples *t*-test. A *P* value was considered significant when less than 0.05. All analyses were performed using SPSS version 27 (IBM, Armonk, NY, USA).

## 3. Results

Ninety-three patients were included in the study sample (51 pre-ERAS and 42 after ERAS). The demographic and clinical data are presented in Table 2, while diagnosis and operative details are shown in Table 3 for each patient. After implementing the ERAS protocol, there was a significant decline in the length of hospital stay while maintaining similar pain control, complication rates, and readmission rates (Table 4). Complications occurred in both groups: postoperative surgical site infection was recorded in both groups, while urinary tract infection (UTI) and deep venous thrombosis (DVT) were limited to the control group. Surgical site infection required readmission for operative irrigation and debridement followed by antibiotics. UTI was treated with intravenous and oral antibiotics, while DVT required high-dose low-molecular-weight heparin to treat. No adverse neurologic outcomes were encountered. The overall incidence of complications was less after implementation of ERAS, but as the number was small, the effect was not found to be statistically significant (14% vs. 5%, *P*=0.31).

## 4. Discussion

The genesis of the ERAS protocols was in the early attempts at reducing the surgical “stress response” in the 1990's literature [12, 13]. The early studies were from other surgical disciplines but still showed great promise in reducing inpatient admission while maintaining or improving outcomes [14–16]. ERAS protocols gained wide acceptance in the past decade, and their implementation in various fields of surgery expanded [17]. The first implementation of ERAS in spine surgery had focused on it as a means to achieve same-day discharge home after minimally invasive lumbar fusions [18, 19]. A significant reduction in the length of stay was observed— for example, up to 73% same-day discharge—while maintaining similar clinical outcomes and pain control. Most of these cases were one-level fusions in low-risk patients, and as such, the value of these studies may apply only to that target population. Later studies reported its use for lumbar decompressions only or spinal surgery in general without making distinctions based on the surgery type [20–22]. The heterogeneous patient sample in these studies limits the clinicians' ability to make clear recommendations for a specific surgical procedure. ERAS implementation in lumbar spinal fusion was first reported by Wang et al. in 2017 [23]. This report had a small sample size—42 patients—and no control group for comparison. The ERAS protocol included local anesthesia, minimally invasive approaches, avoidance of urinary catheterization, multimodal analgesia—excluding nonsteroidal anti-inflammatory medications—and early mobilization. One to two-level fusions were performed only, and the length of stay was 1.29 (range 1–5). Bradywood et al. published their study the same year. They were the first to provide a control group of pre-ERAS patients [24]. Their perioperative protocol was methodologically sound, evidence-based, multidisciplinary, and serially audited for quality improvement purposes. They did not elaborate on the details of their perioperative analgesia protocol but did mention that it was multimodal, standardized, and narcotics were used in a “judicious” way. Their series—a study sample of 244 patients vs. a control of 214—showed a marginal reduction in length of stay while maintaining the same pain control. They did not compare complication rates, but readmission rates were similar between the groups. Smith et al. published their case-control study in 2019 [25]. Their series is limited to short segment fusions and did not include any patients who had previous lumbar surgery. Their ERAS protocol is similar to the other studies but did include nonsteroidal anti-inflammatory medications in their immediate postoperative analgesia protocol. Length of hospital stay was not affected by the ERAS protocol implementation. Still, pain scores consistently were less from postoperative day one to day three despite that opioid use was significantly reduced in the ERAS group (5% vs. 22%). This comes in contradiction to all other series, including our own. The explanation provided was that compliance to the ERAS protocol was “poor.” Kilic et al. published their case-control study in 2020, which included data on cost reduction after ERAS implementation in 88 patients undergoing various lumbar surgeries [26]. Applying a standard ERAS protocol, their results showed a significant decrease in blood loss, transfusion rates, time to the first mobilization, and length of stay. This is comparable to our findings. Notable is the exclusion of patients having high anesthetic risk, which could have biased the results towards ERAS. Complication and readmission rates were not affected. The cost reduction was also substantial in general, except for the cost of the surgery itself, which was unchanged. Two published reports were published in 2021, both reporting exclusively on ERAS implementation in spine deformity surgery. Fletcher et al. published their short-term outcomes after ERAS for spine deformity [27]. Their data—collected prospectively—showed significantly surgical time, blood loss, and length of stay, while pain control was unchanged. The traditional care group had a larger deformity and required more extensive surgery. As such, their results should be interpreted with caution with regard to the target patient population. Kim et al. also reported on ERAS implementation in spine deformity surgery, comparing it to a historical cohort [28]. The two groups were comparable when it came to demographic data and the magnitude of the deformity using various metrics. Blood loss was significantly less in the ERAS group (1437 ml vs. 920 ml), while the length of stay was also reduced from 7.3 days to 4.5 days. Medical complications were reduced from 30% to 10%, while surgical complication rates were identical in the two groups.

## 5. Conclusion

This study presents the initial reports on the first stage of ERAS implementation in our institution. Our results were comparable with other published series, with a reduction in the length of admission while maintaining or improving clinical outcomes. However, being a pilot study, it has several shortcomings. For example, the sample size was small, and the data were collected retrospectively. Also, it lacked any general-health or procedure-specific outcome scores. Starting January 2021, the final ERAS protocol will be implemented in our institution: it includes same-day admission for all cases, prospective SF-36, and Oswestry Disability index data collection for all cases and follow-up for two years.

## Figures and Tables

**Table 1:** ERAS protocol.

Preoperative measures	Intraoperative measures	Postoperative measures
Patient and family education	Blood-loss prevention	Analgesia
(i) By multidisciplinary team (nursing, physicians, and allied healthcare staff)	(i) Wider use of tranexamic acid and assurance of normothermia	(i) Parenteral multimodal analgesia: IV paracetamol (1 gm q6h) and IV morphine 0.1 mg/kg PRN for up to 24 hours
(ii) Clear plan for perioperative care, operative protocol, and postoperative management	(ii) Wider use of bipolar cautery, topical hemostatic agents, and less-invasive posterior approaches	(ii) Alternatively: patient-controlled analgesia
(iii) Discharge planning on the day of admission and outpatient follow-up care fully disclosed	(iii) Discourage use of subfascial drains	(iii) Nausea prevention: metoclopramide or ondansetron
(iv) Patient is fully aware of the target of 3 days or less for most cases		(iv) Once the patient is taking well orally, can be switched to oral analgesics (tramadol 50 to 100 mg q8h, paracetamol 1 gm q8h, and gabapentin 300 mg q8h)
		Early mobilization
		(i) Discontinuation of urinary catheter 6 AM on postoperative day 1
		(ii) Mobilization out of early on postoperative day 1
	Thromboembolic prophylaxis	Nutrition
	(i) Routine application of pneumatic compression devices intraoperatively	(i) Early cessation of parenteral fluid and oral feeding
	(ii) High-risk patients receive chemical prophylaxis	(ii) Routine use of stool-softening agents
	Infection prevention	Discharge plan
	(i) Thorough irrigation throughout the procedure	(i) Discharge medications and outpatient appointment prepared for all early morning on postoperative days 2-3
	(ii) Local application of vancomycin powder	(ii) Patient and family education regarding activities, use of braces, and medications prior to discharge by the multidisciplinary team

**Table 2:** Demographic and operative data in both groups with *P* values.

	Pre-ERAS	ERAS	*P* value
Age (SD)	46 (18)	49 (19)	0.48
Sex, *n* (%)			0.455
Male	17 (33)	11 (26)	
Female	34 (67)	31 (74)	
ASA, *n* (%)			0.116
ASA I	16 (31)	19 (45)	
ASA II	24 (47)	18 (43)	
ASA III	11 (22)	5 (12)	
Procedure type, *n* (%)			0.505
Degenerative	36 (71)	34 (81)	
Deformity	15 (29)	8 (19)	
Number of levels (SD)	3.9 (4)	2.95 (3)	0.634
LOS in minutes (SD)	276 (97)	275 (80)	0.961
EBL in ml (SD)	448 (288)	320 (158)	0.032
Drain placement, *n* (%)	25 (49)	12 (29)	0.045

LOS: length of surgery. EBL: estimated blood loss.

**Table 3:** Diagnosis and surgical management of all patients.

Pre-ERAS							Post-ERAS						
Age	Sex	Diagnosis	Procedure	Number of levels	Levels	Complications	Age	Sex	Diagnosis	Procedure	Number of levels	Levels	Complications
72	M	DLS	PLF	1	L4-L5	—	18	F	Scoliosis	PF	11	T3-L2	—
30	M	Scoliosis	PF	13	T3-L4	—	20	F	Scoliosis	PF	6	T9-L3	—
24	F	Scoliosis	PF	8	T8–L4	—	53	F	LS	TLIF	1	L4-L5	—
43	F	DLS	PLF	2	L3–L5	—	18	M	Scoliosis	PF	11	T3-L2	—
54	M	LS	TLIF	1	L4-L5	—	76	F	DLS	PLF	3	L3–L5	—
74	F	DLS	PLF	4	L2-S1	UTI	58	M	DLS	PLF	1	L4-L5	—
22	F	Scoliosis	PF	12	T3-L3	—	38	M	LS	TLIF	1	L4-L5	—
28	F	DLS	PLF	1	L4-L5	—	39	F	DLS	PLF	1	L4-L5	—
32	F	LS	TLIF	1	L5-S1	—	68	F	LS	TLIF	1	L3-L4	—
62	F	Scoliosis	PF	8	T10-S1	DVT	48	F	DLS	PLF	1	L4-L5	SSI
24	M	Scoliosis	PF	10	T3-L1	—	76	M	LS	TLIF	1	L4-L5	—
40	F	DLS	PLF	2	L4-L5	—	58	F	DLS	PLF	2	L3–L5	—
62	F	DLS	PLF	2	L3–L5	—	51	M	LS	TLIF	2	L4-S1	—
59	F	LS	TLIF	1	L4-L5	—	73	F	LS	TLIF	1	L4-L5	—
71	M	LS	TLIF	1	L4-L5	—	54	F	DLS	PLF	1	L4-L5	—
29	M	Scoliosis	PF	15	T2–L5	—	44	M	LS	TLIF	1	L4-L5	—
58	F	LS	TLIF	1	L5-S1	SSI	80	M	DLS	PLF	1	L4-L5	—
36	M	DLS	PLF	1	L4-L5	—	20	F	Scoliosis	PF	11	T3-L2	—
24	F	Scoliosis	PF	9	T4-L1	—	18	F	Scoliosis	PF	11	T3-L2	—
23	M	Scoliosis	PF	13	T2-L3	—	77	F	DLS	PLF	1	L4-L5	—
56	F	DLS	PLF	2	L3–L5	—	18	F	Scoliosis	PF	12	T3-L3	—
60	F	DLS	PLF	1	L4-L5	—	18	F	Scoliosis	PF	7	T8-L3	—
61	F	LS	TLIF	1	L4-L5	—	57	F	LS	TLIF	1	L4-L5	SSI
57	M	LS	TLIF	1	L5-S1	—	60	F	DLS	PLF	2	L3–L5	—
48	F	Scoliosis	PF	11	T7-S1	—	37	F	DLS	PLF	1	L4-L5	—
55	M	Scoliosis	PF	11	T3-L3	—	73	F	LS	PLF	2	L3–L5	—
39	F	DLS	PLF	1	L4-L5	—	68	F	DLS	PLF	1	L3-L4	—
29	F	DLS	PLF	1	L4-L5	—	18	M	DLS	PLF	2	L4-S1	—
55	M	LS	TLIF	2	L4-L5	—	55	F	LS	TLIF	1	L4-L5	—
50	F	LS	TLIF	1	L5-S1	—	53	M	DLS	PLF	2	L4-S1	—
27	M	DLS	PLF	2	L3–L5	—	52	F	DLS	PLF	2	L3–L5	—
62	F	DLS	PLF	1	L4-L5	SSI	60	F	DLS	PLF	1	L4-L5	—
44	F	DLS	PLF	1	L3-L4	—	58	F	LS	TLIF	2	L4-S1	—
53	F	LS	TLIF	2	L5-S1	—	53	F	DLS	PLF	3	L4-S1	—
51	F	LS	TLIF	2	L5-S1	—	38	F	LS	TLIF	1	L5-S1	—
53	F	DLS	PLF	1	L4-L5	UTI	35	F	LS	TLIF	1	L4-L5	—
39	F	DLS	PLF	1	L3-L4	—	36	M	LS	TLIF	2	L5-S1	—
78	M	DLS	PLF	2	L3–L5	—	63	F	DLS	PLF	2	L4-S1	—
39	F	DLS	PLF	2	L4-S1	—	38	M	LS	TLIF	1	L4-L5	—
66	F	LS	PLF	2	L4-S1	—	53	F	Scoliosis	PF	9	T9-S1	—
66	F	DLS	PLF	2	L3–L5	—	39	F	DLS	PLF	2	L3–L5	—
37	F	DLS	PLF	1	L4-L5	—	75	F	DLS	PLF	1	L4-L5	—
27	F	LS	TLIF	1	L5-S1	—							
62	F	LS	TLIF	1	L4-L5	—							
18	M	Scoliosis	PF	8	T8-L4	—							
64	F	Scoliosis	PF	8	T10-S1	SSI							
33	M	LS	TLIF	1	L5-S1	—							
18	F	Scoliosis	PF	9	T4-L1	—							
18	M	Scoliosis	PF	7	T9-L4	—							
89	M	DLS	PLF	2	L3–L5	SSI							
20	F	Scoliosis	PF	5	T10-L3	—							

Sex: M = male, F = female DLS: degenerative lumbar stenosis, LS: lumbar spondylolisthesis, S: scoliosis PLF: posterolateral fusion, TLIF: transforaminal lumbar interbody fusion, PF: posterior fusion SSI: surgical site infection, UTI: urinary tract infection, DVT: deep vein thrombosis.

**Table 4:** Postoperative data in both groups with *P* values.

	Pre-ERAS	ERAS	*P* value
Day when urinary catheter removed, *n* (%)			0.001
Day 1	8 (16)	28 (67)	
Day 2	38 (75)	11 (26)	
Day 3	4 (8)	2 (5)	
Day 4	1 (2)	1 (2)	
Day when mobilized out of bed, *n* (%)			0.001
Day 1	13 (25)	28 (67)	
Day 2	34 (67)	12 (29)	
Day 3	3 (6)	1 (2)	
Day 4	1 (2)	1 (2)	
Day when switched to oral analgesics, *n* (%)			0.603
Day 1	39 (77)	34 (81)	
Day 2	12 (23)	8 (19)	
Length of hospital stay in days (SD)	5.9 (3.3)	4.2 (1.5)	0.002
Length of hospital stay, *n* (%)			0.001
Three days or less	4 (8)	20 (48)	
Four days or more	47 (92)	22 (52)	
Pain score on DOD (SD)	2 (1.4)	1.6 (1.3)	0.115
PRN analgesic use on DOD, *n* (%)			0.002
None	20 (39)	30 (71)	
Mild	30 (59)	12 (29)	
Moderate	1 (2)	0	
Glycemic control on DOD, *n* (%)			0.49
Controlled	15 (29)	14 (34)	
Borderline	1 (2)	1 (2)	
Uncontrolled	0	1 (2)	
Not diabetic	35 (68)	26 (62)	
Complication rates, *n* (%)	7 (14)	2 (5)	0.31
Readmission rate, *n* (%)	4 (8)	2 (5)	0.547

DOD: day of discharge.

## Data Availability

The data used to support the findings of this study are available from the corresponding author upon request.
